# Performance of BDS-3: Measurement Quality Analysis, Precise Orbit and Clock Determination

**DOI:** 10.3390/s17061233

**Published:** 2017-05-28

**Authors:** Xin Xie, Tao Geng, Qile Zhao, Jingnan Liu, Bin Wang

**Affiliations:** 1 GNSS Research Center, Wuhan University, Wuhan 430079, China; xiexin@whu.edu.cn (X.X.); zhaoql@whu.edu.cn (Q.Z.); jnliu@whu.edu.cn (J.L.); 2 Shanghai Astronomical Observatory, Chinese Academy of Sciences, Shanghai 200030, China; binw@shao.ac.cn

**Keywords:** BeiDou, multipath combination, code pseudo-range, precise orbit determination, clock stability

## Abstract

Since 2015, China has successfully launched five experimental BeiDou global navigation system (BDS-3) satellites for expanding the regional system to global coverage. An initial performance assessment and characterization analysis of the BDS-3 is presented. Twenty days of tracking data have been collected from eleven monitoring stations. The tracking characteristics and measurement quality are analyzed and compared with the regional BDS (BDS-2) in terms of observed carrier-to-noise density ratio, pseudo-range multipath, and noise. The preliminary results suggest that the measurement quality of BDS-3 outperforms the BDS-2 for the same type of satellites. In addition, the analysis of multipath combinations reveals that the problem of satellite-induced code biases found in BDS-2 seems to have been solved for BDS-3. Precise orbit and clock determination are carried out and evaluated. The orbit overlap comparison show a precision of 2–6 dm in 3D root mean square (RMS) and 6–14 cm in the radial component for experimental BDS-3 satellites. External validations with satellite laser ranging (SLR) show residual RMS on the level of 1–3 dm. Finally, the performance of the new-generation onboard atomic clocks is evaluated and results confirm an increased stability compared to BDS-2 satellite clocks.

## 1. Introduction

Since the late 20th century, China has been working on the development of its own satellite navigation system known as BeiDou Navigation Satellite System (BDS). The development has three phases: the demonstration system (BDS-1), the regional system (BDS-2), and the global system (BDS-3) [1]. BDS-1 was declared to be operational at the mid of 2003 with two geostationary satellites and a backup satellite [2]. As a multi-function system, BDS-1 provided users in China with both positioning and short message communication services. BDS-2 has been providing official positioning, navigation and timing (PNT) services over the Asia-Pacific area since 27 December 2012 [3]. The constellation of BDS-2 consists of 14 satellites, five geostationary orbit (GEO), five inclined geostationary orbit (IGSO), and four medium Earth orbit (MEO), transmitting triple-frequency signals centered at B1 (1561.098 MHz), B2 (1207.14 MHz), and B3 (1268.52 MHz). The BDS-3 is expected to complete the constellation deployment with the launching of 35 satellites (five GEOs, three IGSOs, and 27 MEOs) by 2020, providing greatly improved services to global users [1,2,3,4].

With the advent of BDS-2, many studies have been carried out to investigate various aspects of BDS-2, including signal characteristics [5], multipath effects [6], precise orbit determination (POD) [7,8,9], performance of satellite clocks [10,11,12], relative positioning [13], and precise point positioning (PPP) [14], etc. Montenbruck, et al. [7] conducted initial assessment of the BDS-2 based on observations of six reference stations, and the 3D root mean square (RMS) values of orbit overlap comparison were 1–10 m. The BDS-2 satellite orbits determined by Shi, et al. [15] have a radial orbit precision of about 10 cm. Ge, et al. [16] also reported radial orbit overlap RMS values of about 1 dm. Zhao, et al. [8] carried out POD of BDS-2 using three day arcs. The 3D overlap precision reaches about 1.8 m for GEO and 0.3 m for IGSO and MEO, and radial overlap precision is better than 0.1 m for all satellites. In addition, the performance of the indigenous clocks of the BDS-2 has been evaluated [10,11], showing Allan deviations roughly 2–3 times larger than the GPS IIF rubidium atomic frequency standard (RAFS) and the Galileo passive hydrogen maser (PHM). When it comes to the BDS signal characteristics, some studies based on the analysis of multipath combination revealed that the systematic code biases, which are absent for other global navigation satellite system (GNSS), are commonly found in BDS-2 IGSO and MEO satellites [17,18,19]. These biases, called satellite-induced code biases [19,20], are elevation-dependent and can vary more than 1 m from horizon to zenith, which would affect BDS precise applications with code measurements, e.g., ambiguity fixing [20] and single-frequency PPP [19], based on the ionosphere-free code-carrier combination.

On 30 March 2015, China launched an experimental BDS-3 satellite into space, named M1S, for its original global navigation and positioning network using a Long March-3C carrier rocket at the Xichang Satellite Launch Center [21]. The launch marked the beginning of expanding the regional system to global coverage. Up to February 2016, five experimental BDS-3 satellites, including two IGSOs (C31, C32) and three MEOs (C33, C34, C35), have completed deployment (Table 1). Currently, four satellites (C31–C34) are in operation and C35 is in a test orbit. For brevity, the BDS-3 henceforth refers to these experimental BDS-3 satellites in this paper. The task of these satellites is to test the new types of the navigation signals and inter-satellite links, as well as provide services as part of the existing BDS-2 constellation. In addition, all BDS-3 satellites are equipped with new RAFSs and PHMs, which build exclusively on Chinese technology [21]. The BDS-3 includes the migration of its civil B1 signal from 1561.098 MHz to a frequency centered at 1575.42 MHz, which is the same as the GPS L1 and Galileo E1 civil signals, and its transformation from a quadrature phase shift keying (QPSK) modulation to a multiplexed binary offset carrier (MBOC) modulation similar to the future GPS L1C and Galileo E1. Xiao, et al. [22] analyzed the navigation signals transmitted by BDS-3 M2S satellite with the help of a 7.3-m high-gain antenna. Chen, et al. [21] evaluated the contribution of inter-satellite link measurement on BDS-3 precise orbit and clock determination and the performance of BDS-3 satellite clock prediction.

In this contribution, we initially assess the characterization and performance of four BDS-3 satellites based on collected GNSS tracking data. The carrier-to-noise density ratio and multipath combinations of BDS-3 observations are firstly investigated and compared with those of BDS-2 satellites to analyze signal characteristics, pseudo-range multipath and errors. Then, precise orbit and clock determination is carried out by jointly processing for BDS-2 and BDS-3 satellites. Orbit results are evaluated by orbit overlap comparison and SLR validation. The performance of the onboard clocks is analyzed and compared with those of BDS-2 satellites by the modified Allan deviations computed using the clock solutions. The paper is organized as follows: In the “GNSS Processing” section, we describe the data collection, processing strategy, and post-fit residuals for BDS-3 precise orbit and clock determination. In the “Result” section, the results of the analysis of measurement quality and validation of orbit accuracy, as well as the assessment of clock performance, are shown. Conclusions and future work are given in the last section.

## 2. GNSS Processing

### 2.1. Data Collection

**Figure 1:**
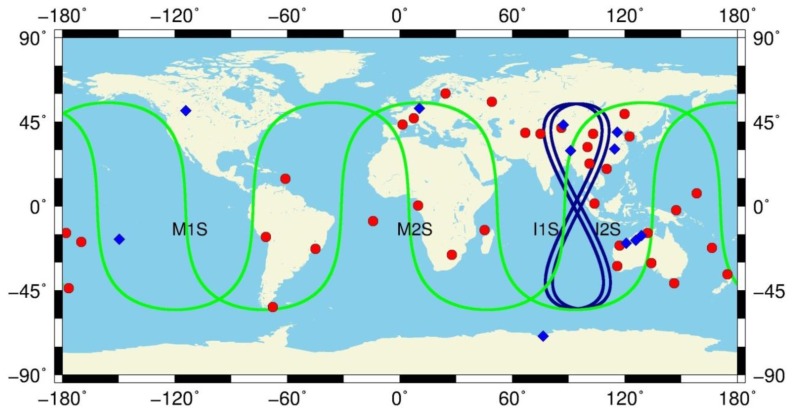
Ground traces of BDS-3 satellites and distribution of the tracking stations used in this study. Stations capable of tracking BDS-3 satellites are marked in blue. The other stations are marked in red.

China has launched the International GNSS Monitoring and Assessment System (iGMAS) [23] to monitor and assess the performance as well as operational status of BDS satellites and to promote compatibility and interoperability among different GNSS systems. BDS-3 observations for the present study are collected by a total of eleven reference stations, among which eight stations are from the iGMAS tracking network, and the other three stations in Australia are from the GA (Geoscience Australia) network. In addition, BDS-2 observations from twenty-six MGEX (Multi-GNSS Experiment) [24] and eight CMONOC (Crustal Movement Observation Network of China) stations are selected for joint POD processing. The orbit and clock results discussed in this paper are based on data for the time period 22 July (DOY 204) until 10 August 2016 (DOY 223). Figure 1 shows the geographic distribution of all GNSS stations and the ground tracks of four BDS-3 satellites. Table 2 lists the information of stations capable of tracking BDS-3 satellites. Since these stations provide only BDS-3 B1 and B3 observations in common with BDS-2, we assess B1 and B3 signal characteristics and use B1 and B3 observations for POD in this paper.

### 2.2. POD Strategy

Orbits of the BDS-2 and BDS-3 satellites, and associated clock solutions, are computed with a modified version of the Positioning And Navigation Data Analyst (PANDA) software package [25], developed at the GNSS Research Center of Wuhan University, China. In this study, the observations from BDS-2 and BDS-3 are jointly processed in a unified processing strategy. We take three consecutive days as one orbit arc and process BDS data of these days to obtain a long arc solution in a batch mode. For each three-day solution, the ionospheric-free linear combinations of B1 and B3 code and phase observations are used to form basic observation equations to eliminate the ionospheric delay. The a priori orbits are taken from the broadcast ephemeris provided by the iGMAS. The BDS-2 and BDS-3 satellite orbit parameters, which include the initial position and velocity, solar radiation pressure (SRP) parameters, satellite clock offsets, station coordinates, receiver clock biases, float ambiguities, and 2-h ZTD (zenith total delay) parameters are estimated together in a weighted least-squares approach. The receiver antenna phase center offset (PCO) and phase center variation (PCV) values are not considered for BDS-3 observations as they are not available. For BDS-2 satellites, the PCO and PCV corrections provided by WHU (Wuhan University) are used resulting in improved performance [23]. For BDS-3 satellites, the PCO corrections are provided by the Operation Control Department, and no PCV corrections are available. For further processing options, see Table 3.

### 2.3. Post-Fit Residuals

**Figure 2:**
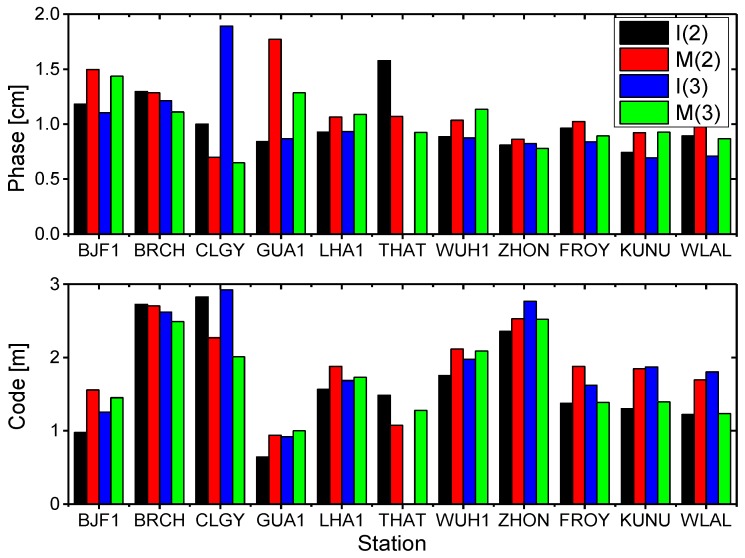
Mean RMS values of the station-specific post-fit residuals of ionosphere-free linear combination of B1 and B3 code and phase observations for BDS-2 and BDS-3 satellites. I(2) and M(2) represent BDS-2 IGSO (C06, C07, C08, C09, C10) and MEO (C11, C12, C14) satellites, and I(3) and M(3) represent BDS-3 IGSO (C31, C32) and MEO (C33, C34) satellites, respectively.

The mean RMS values for the station-specific post-fit residuals of ionosphere-free linear combination of B1 and B3 code and phase observations from the POD parameter estimation are shown in Figure 2. The station THAT (Table 2) cannot observe BDS-3 IGSO (C31 and C32) satellites. The mean phase residuals for all stations are about 1.01 cm, 1.13 cm, 0.99 cm, and 1.01 cm for BDS-2 IGSO, BDS-2 MEO, BDS-3 IGSO, and BDS-3 MEO satellites, respectively. The mean code residuals for all stations are about 1.66 m, 1.86 m, 1.94 m, and 1.69 m for BDS-2 IGSO, BDS-2 MEO, BDS-3 IGSO, and BDS-3 MEO satellites, respectively. We can see that the phase and code post-fit residuals of BDS-2 and BDS-3 are nearly the same level. It is noted that these errors are amplified by a factor of about three when forming the ionosphere-free linear combination, compared to the single-frequency measurement. In the next section, we will further analyze pseudo-range errors (multipath and noise) by the multipath combination.

## 3. Results

In this section, we present the results of measurement quality analysis, including carrier-to-noise density ratio (C/N0), multipath combination, orbit accuracy validation, and clock performance assessment for four BDS-3 satellites. For comparison, some results of BDS-2 satellites are also shown.

### 3.1. Measurement Quality Analysis

#### 3.1.1. Carrier-to-Noise Density Ratio

**Figure 3:**
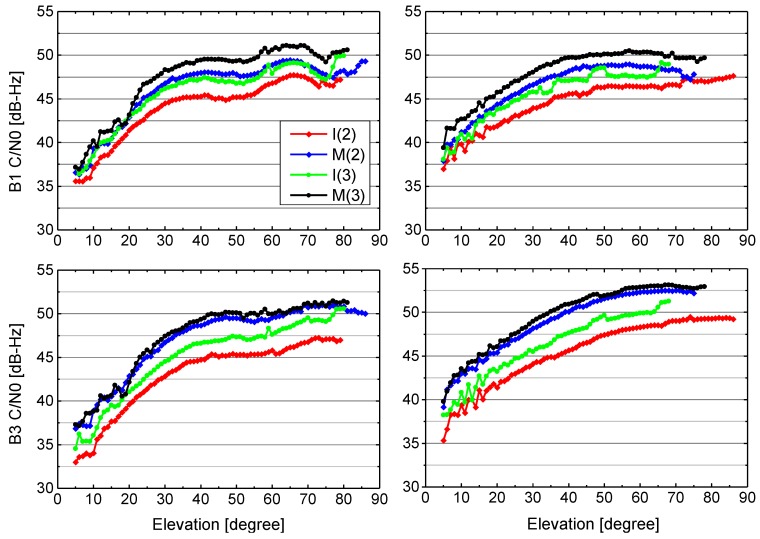
Mean C/N0 values as a function of elevation for BDS-2 and BDS-3 IGSO and MEO satellites signals at site BJF1 equipped with GMR-4011 receiver with LEIAR25.R4 antenna (left) and site KUNU equipped with POLARX5 receiver with JAVRINGANT_DM antenna (right) during DOY 204-221, 2016. The upper and bottom panels show the results of B1 and B3 frequencies, respectively.

The C/N0 ratio given by GNSS receiver is a result of signal gain and loss along the complete transmitting and receiving chain [5]. The whole chain consists of the electronic circuit and antenna of the satellite, the signal path, and the antenna and the electronic circuit of the receiver. The observed C/N0 values of BDS-2 and BDS-3 signals at sites BJF1 and KUNU equipped with the GMR-4011 and POLARX5 receivers are depicted as a function of elevation in Figure 3. Eighteen days of tracking data between DOY 204 and DOY 221 (2016) are used to produce the results. We first group all C/N0 values according to their corresponding elevations separately for B1 and B3 signal of all BDS-2 IGSO, BDS-2 MEO, BDS-3 IGSO, BDS-3 MEO satellites. Then, all C/N0 values within an elevation range of 1° are classified into one group. Finally, a mean value of C/N0 is computed and shown for each elevation group.

From the figure, BDS-2 and BDS-3 signals from these two stations exhibit generally similar elevation-dependent C/N0 characteristics, from about 35 dB-Hz (at the low elevation) to about 50 dB-Hz (at the high elevation). MEO satellites are generally tracked with a 2–4 dB-Hz higher C/N0 values than the IGSO satellites, which are related to the smaller orbital radius. Furthermore, it is obvious that the B1 signal of BDS-3 IGSO and MEO satellites has approximately 1–2.5 dB-Hz higher C/N0 values than the corresponding BDS-2 IGSO and MEO satellites, respectively. For B3 signal, the C/N0 values of BDS-3 IGSO are higher by about 1–4 dB-Hz than BDS-2 IGSO, and BDS-3 MEO are higher by about 1–2 dB-Hz than BDS-2 MEO. In general, the signal strength of BDS-3 signals exceeds the values of the BDS-2 for the same satellite type on the each frequency.

#### 3.1.2. Multipath Combination

The multipath (MP) combination is a measure of the combined results of multipath, receiver noise, and bias variations between pseudo-range and carrier-phase measurements. The combination is constructed using a single-frequency code measurement and dual-frequency phase measurements of a pass that one receiver is continuously tracking for one GNSS satellite without cycle slips [31]. The constant biases, such as ambiguities, hardware delay in satellite and receiver are eliminated by averaging the MP series of a pass. The residual series are dominated by the multipath and noises of pseudo-range measurements since the carrier phase multipath and noises are much smaller in magnitude.

**Figure 4:**
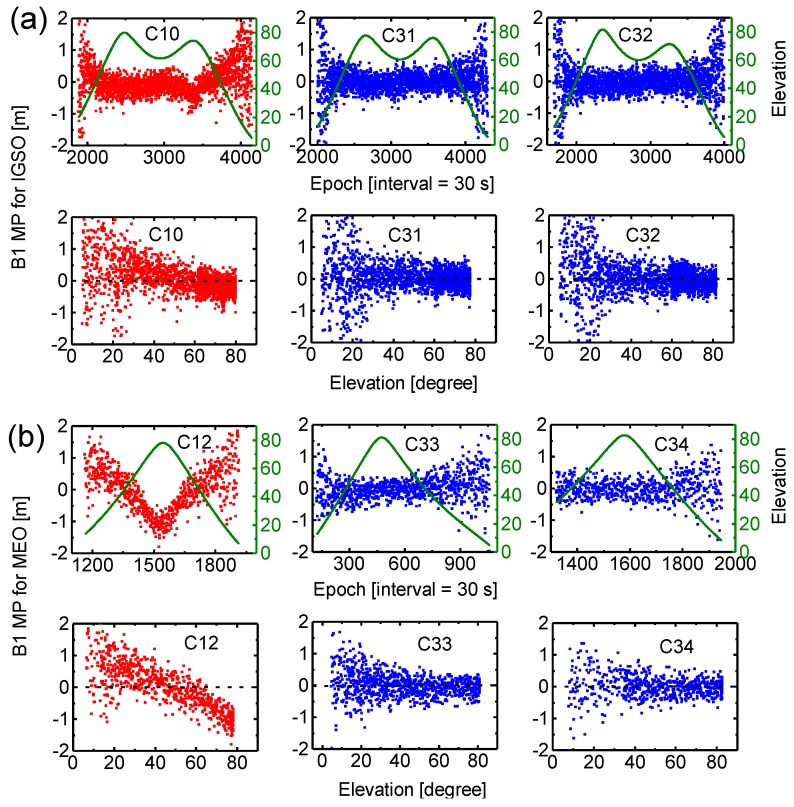
MP combinations of BDS-2 (red) and BDS-3 (blue) B1 frequency as a function of time and a function of elevation angle for IGSO (**a**), and MEO (**b**) satellites at station BJF1. The olive lines represent corresponding satellite elevation.

**Figure 5:**
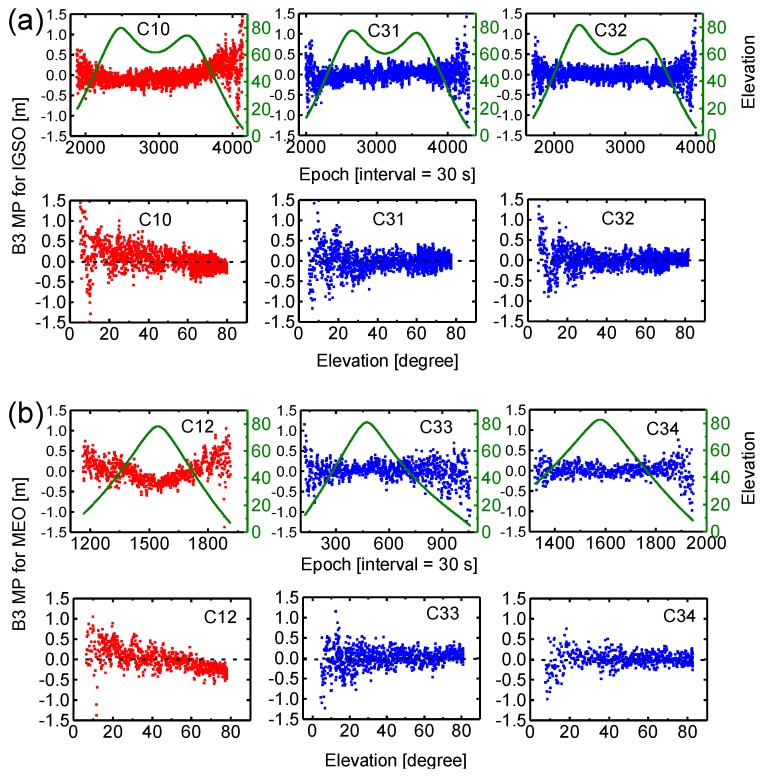
MP combinations of BDS-2 (red) and BDS-3 (blue) B3 frequency as a function of time and a function of elevation angle for (**a**) IGSO, and (**b**) MEO satellites at station BJF1.

Figure 4 shows MP time series of the B1 signal for BDS-2 (C10, C12) and BDS-3 (C31, C32, C33, C34) satellites during a pass as a function of time and a function of elevation angle, sampled at 30 s, at station BJF1 according to different satellite types, i.e., IGSO (a) and MEO (b) satellites. In general, the continuous tracking duration is about 18–20 h and 7–8 h for IGSO and MEO satellites. Note that C34 satellite tracking data of about one hour at the beginning is lost. As expected, signals observed at low elevation angles are affected more severely by multipath conditions. Moreover, we can see from MP series that BDS-2 satellites (red) contain additional systematic biases (drifts), namely, the satellite-induced code biases, which are obviously elevation-dependent. The code bias variations could reach up to about 1 m at high elevation angles, particularly for BDS-2 MEO satellite. These results of BDS-2 MP combinations are very consistent with those discussed in Hauschild, et al. [5] and Wanninger and Beer [19], while for BDS-3 satellites (blue), these code biases found in BDS-2 signals have been greatly reduced, comparable to those of other GNSS systems [32]. Figure 5 shows the results for MP combinations of the B3 signal for BDS-2 and BDS-3 satellites. We can see that the multipath errors and noise level of B3 signal are smaller than those of B1 for each satellite. Similarly, there are almost no code biases in BDS-3 measurements for B3 signal.

**Figure 6:**
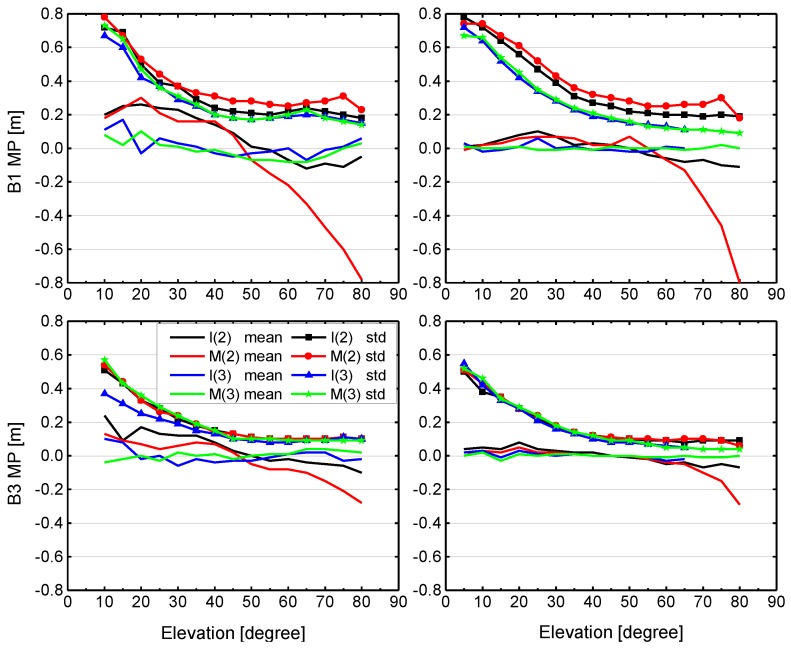
Mean and standard deviation (std) of MP combinations as a function of elevation for BDS-2 and BDS-3 IGSO and MEO satellites signals at stations BJF1 (left) and KUNU (right) during DOY 204-221, 2016. The upper and bottom panels show the results for B1 and B3, respectively. I(2) and M(2) represent BDS-2 IGSO (C06, C07, C08, C09, C10) and MEO (C11, C12, C14) satellites, and I(3) and M(3) represent BDS-3 IGSO (C31, C32) and MEO (C33, C34) satellites.

In order to further analyze and compare pseudo-range noise, multipath errors and satellite-induced code biases, we plot results of the MP combination for B1 and B3 measurements for two individual stations, as shown in Figure 6. All values of MP combination are grouped according to their corresponding elevations, and the mean and standard deviation are computed for each satellite type (IGSO and MEO). The results for the CETC-54 GMR-4011 receiver (The 54th Research Institute of China Electronics Technology Group Corporation, Shijiazhuang, China) at site BJF1 are depicted in the left panels, and the SEPT POLARX5 receiver (Septentrio, Leuven, Belgium) at site KUNU is shown in the right panels. Again, observations from the same test period between DOY 204 and DOY 221 (2016) are used. We can see that the variation of the mean and standard deviation with elevation reveals a similar characteristic for the two stations. For the mean values, BDS-3 exhibits a typical stochastic error (zero-mean), whereas an obvious systematic bias can be observed in BDS-2 for each receiver and each frequency. The elevation-dependent bias for B1 signal is more pronounced than B3, and IGSO is smaller than MEO. The standard deviation does not include the systematic bias component. For B1 signal, a standard deviation of about 0.8 m at low elevations angles and about 0.2 m close to zenith can be seen for BDS-2 and BDS-3. It becomes obvious that the multipath and noise for the B1 signal are higher compared to B3, which exhibits a standard deviation of about 0.5 m at low elevations angles and about 0.1 m at high elevation angles. Moreover, the standard deviations of B1 signal for BDS-3 are, on whole, slightly smaller than BDS-2 for the same satellite type at these two sites. Most important of all, the results of Figure 4, Figure 5 and Figure 6 indicate that the new-generation BeiDou satellites have improved satellite-internal hardware device design to avoid the problem of code biases like for BDS-2, which would provide better performance.

### 3.2. Orbit Validation

**Figure 7:**
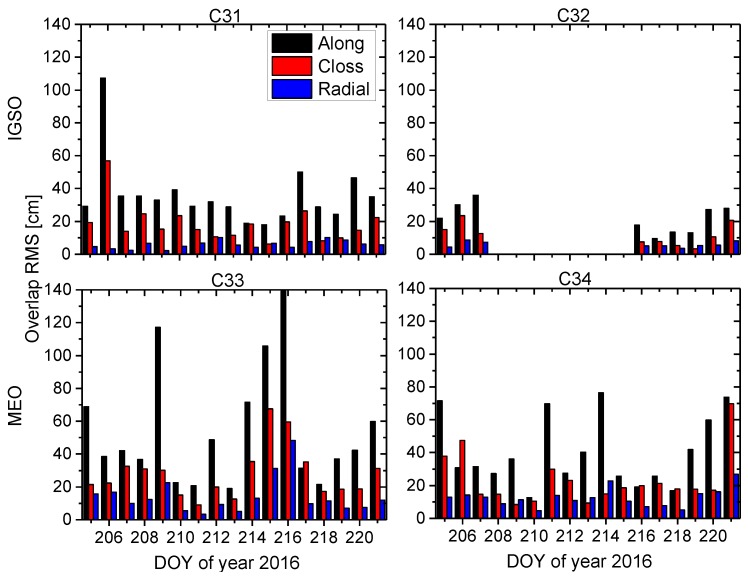
RMS values of 48-h orbit overlap errors for four BDS-3 satellites in along-track, across-track, and radial directions during the period of the experiment.

We assess BDS orbit quality through both internal consistency and external validation. For internal consistency, the direct comparison of satellite positions in the overlapped time span from different orbit solutions is utilized in this study. For any two adjacent three-day solutions shifted by one day, there are 48-h orbit overlap errors. Figure 7 shows the RMS values of 48-h orbit overlap errors of every orbital arc during the experiment period for BDS-3 satellites C31, C32, C33, and C34 in along-track, across-track, and radial directions. As an unfortunate exception, the C32 satellite was in a test orbit from DOY 210 to 214 in 2016 and, therefore, no results of orbit validation can be shown. The RMS differences between the days are quite large (Figure 7), which is because of serious loss of data at some stations, especially for site THAT (Table 2) having only two days of tracking data available. In addition, the mean RMS values of orbit overlap comparison for each BDS-3 satellite and each type of BDS-2 IGSO and MEO satellite are listed in Table 4. From the results of orbit overlap comparison in Figure 7 and Table 4, the RMS values of the along-track component are the largest for all satellites and the radial component has the smallest RMS values due to the observation geometry. The averaged 3D RMS values are 33.4 cm and 55.5 cm, and the averaged RMS values in the radial component reach 5.9 cm and 13.4 cm for BDS-3 IGSO and MEO satellites, respectively. BDS-3 IGSO satellite shows smaller orbit overlap errors than MEO satellite, simply because there are better spatially-distributed BDS-3-capable tracking stations used in the POD and ground traces of satellites (refer to Figure 1).

It can be seen from Table 4 that the RMS values of BDS-2 IGSO and MEO satellites are smaller than BDS-3 corresponding type of satellites, which may be due to the higher number and better spatial distribution of tracking stations for BDS-2. To validate this assumption, we conducted BDS-2 and BDS-3 POD in a unified processing strategy using only the stations listed in Table 2, which are capable of tracking BDS-2 and BDS-3 satellites simultaneously. The results of orbit overlap comparison are listed in Table 5. We can see that the averaged RMS values are larger than the corresponding results in Table 4, especially for BDS-2. This indicates that the insufficient number and poor spatial distribution of the tracking stations are the main reasons why BDS-3 POD precision is relatively poor, as listed in Table 4. However, the averaged RMS values for BDS-2 MEO are still smaller than the BDS-3 MEO as listed in Table 5. This might be due to a lesser number of available observations for C33 and C34 satellites.

For external validation, satellite laser ranging (SLR) residuals provide the opportunity to assess estimated orbit accuracy as they are based on an independent observation technique. BDS-3 satellites C31, C32, C33, and C34 are equipped with laser retro-reflectors and tracked by several SLR stations coordinated by the International Laser Ranging Service (ILRS) [33]. Since the length of a POD arc is three days, only the orbital solutions of the middle day are used for validation. For the time period considered in this study, only a very limited number of normal points (NPs) for BDS-3 satellites are available: one SLR station tracked C31 (Mt Stromlo: four NPs), two SLR station tracked C32 (Shanghai, Changchun: 20 NPs), three stations for C33 (Herstmonceux, Matera, Monument Peak: 21 NPs), and two stations for C34 (Shanghai, Yarragadee: six NPs). One has to keep in mind that the SLR validation primarily assesses the radial component of satellite orbit. The mean, standard deviation (STD), and RMS of the SLR residuals for the four BDS-3 satellites are listed in Table 6. The overall RMS values for C31, C32, C33, and C34 are 19.6 cm, 27.1 cm, 20.7 cm, and 10.9 cm, respectively, which are slightly larger than that of overlap comparison in the radial component.

### 3.3. Clock Performance

The onboard atomic frequency standard is a key component of a GNSS satellite. The stability of the satellite clock affects the accuracy of the clock offset modeling and prediction of broadcast information and, thus, directly limits the user positioning and timing accuracy. Therefore, a high predictability of the onboard frequency standards is required to overcome this limitation and enable an extended validity of clock correction parameters. Since the early days of BDS, an improved accuracy of the onboard clocks has been striven for with every new generation of satellites. Compared to BDS-2, the primary frequency standard of BDS-3 satellites is based on the new RAFS and PHM made by Chinese technology. During the experiment period, the new rubidium clocks are active as the primary frequency standards in the C31, C33, and C34 satellites, and a PHM is active in satellite C32.

**Figure 8:**
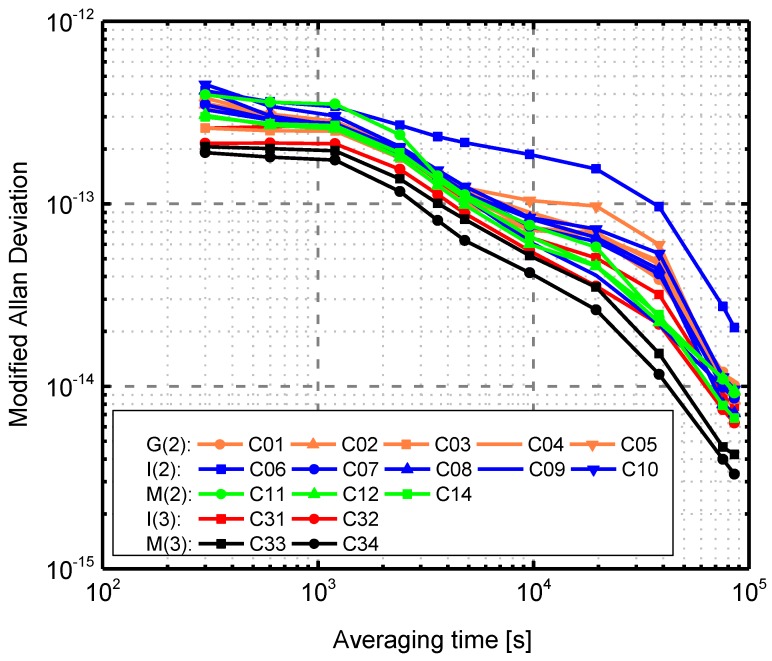
Modified Allan deviation of BDS-3 (C31–C34) satellite clocks for the time period DOY 204–223 (2016). For comparison purposes, the performance of the BDS-2 satellite clocks is also shown in the plot. G(2), I(2) and M(2) represent BDS-2 GEO (C01–C05 ), IGSO (C06–C10) and MEO (C11, C12, C14) satellites, and I(3) and M(3) represent BDS-3 IGSO (C31, C32) and MEO (C33, C34) satellites, respectively.

To assess the performance of the BDS-3 onboard clocks in terms of frequency stability, modified Allan deviations (MADEV) computed using the 300 s clock estimates obtained from the above-mentioned precise orbit and clock determination are shown in Figure 8. Before the computation of MADEV, the outliers, clock jumps and frequency steps in the satellite clock time series were detected and preprocessed [34]. In all cases, the hydrogen maser at site XATT located in Xi’an (China) has been selected as a reference for the clock estimation. For BDS-2 satellites, at an integration time of 1000 s, the values of MADEV for all satellites are about 2–$4\times 10^{-13}$. At an integration time of 10,000 s, the values are about 6–$10\times 10^{-14}$. These results are consistent with those given in Wang, et al. [10] on the magnitude and relative size of each satellite, except for the C02 satellite, which is known to suffer clock adjustments. Note that the results of BDS-2 shown here are slightly worse than those of Montenbruck, et al. [7] and Steigenberger, et al. [11], likely due to the quality of the reference clock, the orbit and clock quality. The degraded clock performance of C06 shown in Figure 8 compared to the other IGSO satellites is also mentioned by Prange, et al. [35].

For BDS-3 satellites, the frequency stability of onboard clocks is better than that of BDS-2 on the whole, particularly for the two MEO satellites C33 and C34. C34 shows the best performance, with a MADEV of about $1.5\times 10^{-13}$ at an integration time of 1000 s, and $4.1\times 10^{-14}$ at an integration time of 10,000 s. In addition, the mean frequency stability of 1000 s, 10,000s, and one day, according to different satellite types, is listed in Table 7. For IGSO, BDS-3 shows an improvement of 19.2%, 38.8%, and 33.3% for frequency stability of 1000 s, 10,000 s, and one day, respectively. For MEO, the corresponding improvement is 36.8%, 30.7%, and 55.0%, respectively. In summary, BDS-3 satellite clocks outperform previous BDS-2 clocks in terms of frequency stability thanks to new RAFS and PHM.

## 4. Conclusions

The transmitted signals of BDS-3 satellites on B1 and B3 frequencies have been tracked by several GNSS monitoring stations from GA and iGMAS network, enabling a meaningful characteristic analysis and performance assessment of this newer generation of the GNSS constellation. We analyzed the observed C/N0 values of BDS-2 and BDS-3 B1 and B3 signals at two sites equipped with different receivers and antennas. The results indicate that the signal strength of BDS-3 exceeds BDS-2. Based on MP combinations, we investigated the multipath effect and receiver noise structure, as well as bias variations between pseudo-range and carrier-phase measurements. Different from other GNSS, the BDS-2 code measurements are affected by satellite-induced biases larger than 1 m from horizon to zenith, which will inevitably affect the BDS-2 included precise applications which involve the code measurements, e.g., ambiguity resolution based on the Hatch-Melbourne-Wübbena (HMW) combination [36,37,38]. Fortunately, these biases do not seem to appear in BDS-3 B1 and B3 code measurements.

The BDS-3 orbit and clock parameters were estimated with a smaller amount of tracking stations, showing that an orbit overlap precision of 2–6 dm in 3D RMS and 6–14 cm in the radial component for four BDS-3 satellites is achievable. With a better spatially-distributed tracking network and less data loss, the accuracy of orbit estimates is expected to be considerably improved, reaching a comparable accuracy with BDS-2 IGSO and MEO satellites on the level of 1–2 dm. The new-generation RAFS and PHM are currently active as primary frequency standards for BDS-3 satellites. We evaluated the performance of the BDS-3 clocks using a modified Allan deviation. The results indicate that the frequency stability of BDS-3 clocks is improved by about 20–50% compared to BDS-2.

The results obtained in this contribution are rather promising. A larger tracking network, in particular a network providing global coverage for the MEO satellites is an important step for the improvement of the BDS-3 orbit and clock solutions. However, there are still many related issues requiring further investigations to achieve better accuracy. First, the PCO and PCV of the BDS-3 satellites should be estimated and refined in the future. Second, the attitude control mechanism and solar radiation pressure model for BDS-3 should be tested and confirmed. In addition, the comparison of BDS-3 clocks performance for both long and short-term stability with the latest generation of RAFS and PHM of other GNSS, such as GPS and Galileo, is considered as an on-going research effort.

## Figures and Tables

**Table 1:** The status of the experimental BDS-3 satellites (February 2016).

Satellite	PRN	Type	Launch Date	Carrier Rocket	Mean Longitude (Inclination)
I1S	C31	IGSO	30 March 2015	Long March-3C	92.8° E (55.5°)
I2S	C32	IGSO	29 September 2015	Long March-3B	96.5° E (55.5°)
M1S	C33	MEO	25 July 2015	Long March-3B	(55.5°)
M2S	C34	MEO	25 July 2015	Long March-3B	(55.5°)
M3S	C35	MEO	1 February 2016	Long March-3C	(55.5°)

**Table 2:** GNSS monitoring stations with BDS-3 tracking capability.

Abb.	Location	Country	Receiver	Antenna
BJF1	Beijing	China	CETC-54 GMR-4011	LEIAR25.R4 LEIT
BRCH	Braunschweig	Germany	CETC-54 GMR-4011	LEIAR25.R4 LEIT
CLGY	Calgary	Canada	CETC-54 GMR-4011	LEIAR25.R4 LEIT
GUA1	Urumqi	China	GNSS_GGR	RINT-8CH CETD
LHA1	Lhasa	China	CETC-54 GMR-4011	NOV750.R4 NOVS
TAHT	Tahiti	France	GNSS_GGR	RINT-8CH CETD
WUH1	Wuhan	China	CETC-54 GMR-4011	LEIAR25.R4 LEIT
ZHON	Antarctica	United Nations	CETC-54 GMR-4011	LEIAR25.R4 LEIT
FROY	Fitzroy Crossing	Australia	SEPT POLARX5	LEIAR25.R3 LEIT
KUNU	Kununurra	Australia	SEPT POLARX5	JAVRINGANT_DM SCIS
WLAL	Wallal	Australia	SEPT POLARX5	LEIAR25.R3 LEIT

**Table 3:** Important options of the GNSS processing for POD.

Item	Models
Basic observable	B1 and B3 code and phase observations of BDS-2 and BDS-3
Modeled observable	Ionospheric-free linear combination
Sampling rate	300 s
Elevation cutoff	10°
Weighting	A priori precision of 0.002 m and 2.0 m for raw phase and code observables, respectively, and elevation-dependent data weighting
Phase wind up	Phase polarization effects applied [26]
Tropospheric delay	Saastamoinen model [27], global mapping function [28], two-hourly ZTD without gradients
Tide displacement	Solid Earth tide, pole tide, ocean tide loading; according to IERS Conventions 2003 [29]
Relativity effect	Considered according to IERS Conventions 2003 [29]
Geopotential	EIGEN_GL04C up to 12 × 12 degree
N-body gravitation	Sun, Moon, and other planets; JPL DE405 ephemeris used
Solar radiation	ECOM 5-parameter model [30]

**Table 4:** Averaged RMS values of 48-h orbit overlap errors for BDS-3 and BDS-2 satellites (unit: cm).

Satellite		Along	Cross	Radial	3D
IGSO	C31	36.1	18.6	5.9	41.1
	C32	21.9	11.8	5.9	25.6
MEO	C33	54.3	28.1	14.2	62.8
	C34	40.4	23.1	12.6	48.2
BDS-2 GEO		224.0	8.1	7.1	224.2
BDS-2 IGSO		17.9	11.2	4.6	21.6
BDS-2 MEO		10.3	6.2	2.8	12.3

**Table 5:** Averaged RMS values of 48-h orbit overlap errors for BDS-2 satellites using only the reference stations with BDS-3 and BDS-2 simultaneous tracking capability (unit: cm).

Satellite		Along	Cross	Radial	3D
IGSO	C31	46.9	38.1	8.3	61.0
	C32	38.7	34.5	7.5	52.4
MEO	C33	53.9	27.2	15.1	62.2
	C34	50.4	37.0	14.1	64.1
BDS-2 IGSO		41.7	29.4	7.2	51.5
BDS-2 MEO		41.4	29.7	9.3	51.8

**Table 6:** SLR residuals for BDS-3 satellites C31, C32, C33, and C34.

Satellite	# NP	Mean (cm)	STD (cm)	RMS (cm)
C31	4	−19.6	0.4	19.6
C32	20	25.0	10.7	27.1
C33	21	9.8	18.3	20.7
C34	6	−3.3	10.4	10.9

**Table 7:** Statistics of frequency stability of BDS-2 and BDS-3 satellite clocks.

MADEV	BDS-2 GEO	BDS-2 IGSO	BDS-2 MEO	BDS-3 IGSO	BDS-3 MEO
1000 s	$2.80\times 10^{-13}$	$3.02\times 10^{-13}$	$2.99\times 10^{-13}$	$2.44\times 10^{-13}$	$1.89\times 10^{-13}$
10,000 s	$8.43\times 10^{-14}$	$9.65\times 10^{-14}$	$6.65\times 10^{-14}$	$5.91\times 10^{-14}$	$4.61\times 10^{-14}$
1 day	$8.94\times 10^{-15}$	$1.02\times 10^{-14}$	$7.98\times 10^{-15}$	$6.80\times 10^{-15}$	$3.59\times 10^{-15}$
